# The role of Nrf2 signaling in parasitic diseases and its therapeutic potential

**DOI:** 10.1016/j.heliyon.2024.e32459

**Published:** 2024-06-14

**Authors:** Mohammadamin Vatankhah, Reza Panahizadeh, Ali Safari, Alireza Ziyabakhsh, Behnam Mohammadi-Ghalehbin, Narges Soozangar, Farhad Jeddi

**Affiliations:** a Zoonoses Research Center, Ardabil University of Medical Sciences, Ardabil, Iran; b Students Research Committee, School of Medicine, Ardabil University of Medical Sciences, Ardabil, Iran; c Department of Genetics and Pathology, School of Medicine, Ardabil University of Medical Sciences, Ardabil, Iran

**Keywords:** Entamoebiasis, KEAP1, Leishmaniasis, Malaria, NRF2, ROS, Schistosomiasis, Toxoplasmosis, Trypanosomiasis

## Abstract

In response to invading parasites, one of the principal arms of innate immunity is oxidative stress, caused by reactive oxygen species (ROS). However, oxidative stresses play dual functions in the disease, whereby free radicals promote pathogen removal, but they can also trigger inflammation, resulting in tissue injuries. A growing body of evidence has strongly supported the notion that nuclear factor erythroid 2-related factor 2 (NRF) signaling is one of the main antioxidant pathways to combat this oxidative burst against parasites. Given the important role of NRF2 in oxidative stress, in this review, we investigate the activation mechanism of the NRF2 antioxidant pathway in different parasitic diseases, such as malaria, leishmaniasis, trypanosomiasis, toxoplasmosis, schistosomiasis, entamoebiasis, and trichinosis.

## Highlights

• Nrf2 is essential for maintaining intracellular redox balance and controlling inflammation.
• Nrf2 has dual roles in parasitic infection, benefiting either the parasite or the host.
• ROS-resistant parasites may be damaged by a strong antioxidant state induced by Nrf2 activation.
• Suppression of Nrf2 expression has therapeutic potential in visceral leishmaniasis.

## 1 Introduction

Parasitic organisms could cause several diseases, affecting numerous people, especially in developing countries. Innate immunity is the first line of non-specific defense against parasites. Many parasites are more susceptible to reactive oxygen species (ROS) related to their hosts. Therefore, the inflammatory response against parasitic microorganisms, which is accompanied by the generation of ROS, is one of the main pillars of innate immunity. Uncontrolled inflammation, by creating an imbalance between oxidants and antioxidants, could cause much damage to the host [1,2]. Oxidative stress elicits apoptosis and oxidative stress-induced diseases, such as neurodegenerative and cardiovascular diseases, accelerated aging, secondary infections, and malignancies. In various infections, it may be possible to prevent oxidant-mediated cytotoxicity and apoptosis in the host cells by increasing antioxidant expression [[3], [4], [5]].

**Fig. 1:**
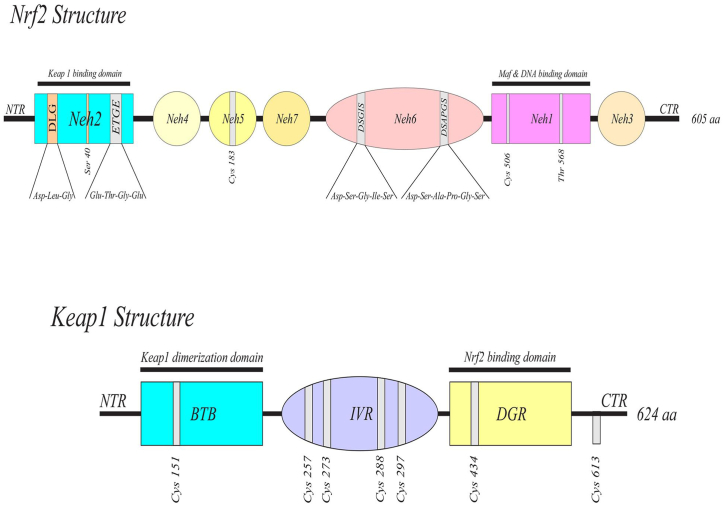
The functional structures of Nrf2 and Keap1.Human NRF2 protein comprises 605 amino acid residues with 7 conserved Nrf2-ECH homology (Neh) domains (Neh1 to Neh7). Neh1 domain has a bZIP motif, which participates in heterodimerization with small MAF protein and in binding to ARE to activate ARE-dependent gene expression. Neh2 is another highly conserved domain, which functions as a negative regulatory element in the transcriptional activity of Nrf2. KEAP1 is a 70-kDa cysteine-rich protein containing 624 amino acids with five domains including, NTR, BTB, IVR, DGR, and CTR. The important cysteine residues on the Keap1, Cys151 for dimerization, and Cys257, Cys273, Cys288, and Cys297 for redox sensing have been shown.DGR domain has several protein contact positions that intermediate the interaction between Nrf2 and Keap1 (the Neh2 domain of Nrf2 interacts with kelch domain of Keap1). ARE: antioxidant response element, BTB: broad complex, tramtrack, and bric-a-brac, CTR: the C-terminal region, DGR: double glycine/Keclch repeats, IVR: intervening region, KEAP1: Kelch-like ECH-associated protein, Neh: Nrf2-ECH homology domain,Nrf2: nuclear factor erythroid 2-related factor 2, NTR: N-terminal region.

**Fig. 2:**
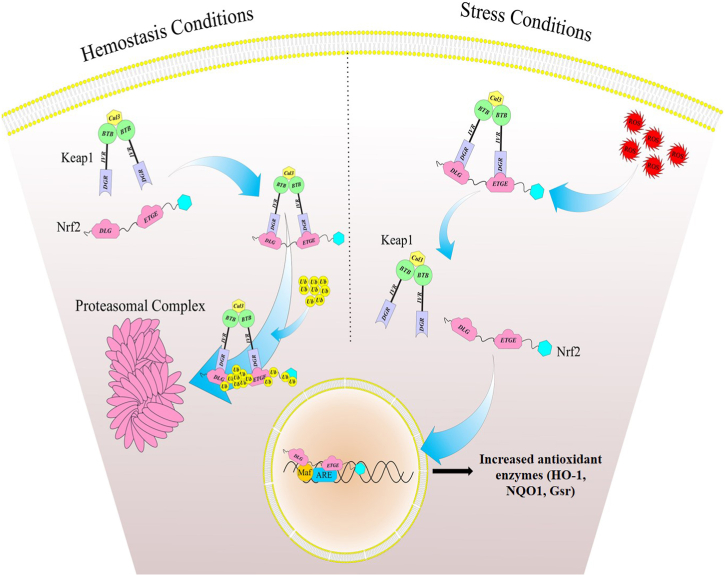
Interaction between Nrf2 and Keap1.Under hemostasis situations, Keap1-Nrf2binding in the cytosol leads to proteasomal degradation of Nrf2. In oxidative conditions, the modification of cysteine residues in KEAP1 protein is followed by the translocation of free Nrf2 to the nucleus. Then Nrf2 binds with the MAF proteins and ARE in the promoter of cytoprotective genes, which resulted in the activation of antioxidant enzymes (HO-1, NQO1, and Gsr). ARE: antioxidant response element, Gsr: glutathione reductase, HO-1: Heme Oxygenase-1, KEAP1: Kelch-like ECH-associated protein, Maf: musculoaponeurotic fibrosarcoma, NQO1: NAD (P) H Quinone Dehydrogenase 1, Nrf2: nuclear factor erythroid 2-related factor 2.

Nuclear factor erythroid 2-related factor 2 (NRF2), as a transcriptional factor, is involved in the regulation of oxidative stress response. Another key molecule in the inflammatory pathway is Kelch-like ECH-associated protein (KEAP1), which is a negative regulator of NRF2 (Fig. 1). As described in our previous study [1], under hemostasis conditions, the binding of KEAP1 to NRF2 in the cytosol leads to the degradation of NRF2. In oxidative stress conditions, the alteration of cysteine residues in KEAP1 leads to the translocation of free NRF2 to the nucleus, where NRF2 is complexed with the MAF proteins and ARE. This complex then binds to the promoter region of antioxidant genes coding cytoprotective antioxidant enzymes such as heme oxygenase 1 (*HO-1*), NAD (P) H Quinone Dehydrogenase 1 (*NQO1*), glutathione reductase (*Gsr*) (Fig. 2) [6,7].

*Nrf2* and its downstream antioxidant genes are essential for the preservation of intracellular redox equilibrium and the control of inflammation. In response to the oxidative stress, more than 200 detoxifying and cytoprotective genes were found to be controlled by *Nrf2* [1,[8], [9], [10]]. This network could be activated by pathogens through different mechanisms, such as the involvement of toll-like receptors, the induction of the *PI3K/Akt* pathway, or endoplasmic reticulum stress. Infections by protozoans like *Leishmania*, *Entamoeba*, *Cryptosporidium*, *Plasmodium*, and *Toxoplasma* spp. result in the modulation of *Nrf2* and a diminished anti-inflammatory and antioxidant pattern [11,12]. It, therefore, seems that the role of *Nrf2* in the pathogenesis of parasitic infections is now beginning to be unveiled. In this study, we reviewed the roles of activation or inhibition of the NRF2 pathway in various parasitic diseases and its therapeutic potential. Table 1 summarizes the therapeutic targeting of the Nrf2 signaling pathway in parasitic diseases.

**Table 1:** Summary of the therapeutic targeting of the Nrf2 signaling pathway in parasitic diseases.

Parasite	Mediator	Mechanism of action	Reference
Visceral leishmaniasis	Miltefosine	Nrf2↓, HO-1↓, GPx↑, SOD↑, ROS↑, IL-10 ↓, TGF-β↓, IL-12↑, TNF-α↑	[[13], [14], [15]]
*L. braziliensis*	Quercetin	Nrf2↑, HO-1↑, IL-10↑, TNF-α↓	[16]
*L. amazonensis*	DHA	Nrf2↓, HO-1↓, ROS↑	[17]
*S. mansoni*	ZLE	Nrf2↑, TGF-β↓, α-SMA↓	[18]
*S. japonicum*	PPI	Nrf2↑, GSH↑, TGF-β↓, Gsta4↑	
*T. gondii*	IOP	Nrf2↑, HO-1↑, GSH↑, SOD↑, TNF-α↓, IL-6↓, IL-1β↓, IFN-γ↓, IL-4↓	[19]
*T. cruzi*	Melatonin	Nrf2↑, NF-κB↓, IL-10↓, IL-4↓, TNF-α↓	[20,21]
*T. cruzi*	BZL	Nrf2↑, GPx↑, GSH↑	[22,23]
*T. cruzi*	SFN	Nrf2↑, GPx↑	[22]
*P. falciparum*	DMF	Nrf2↑, TNF-α↓, IL-6↓,	[24]
*P. berghei*	Lovastatin	Nrf2↑, HO-1↑, TNF-α↑, IFN-γ↓,	[25]
*P. berghei*	VEGF–lovastatin	Nrf2↑, HO-1↑, TNF-α↓, IFN-γ↓	[25]
*P. berghei*	L. Inermis	Nrf2↑, GP_X_↑, SOD↑, GSH↑, CAT↑, GST↑, TNF-α↓, *iNOS*↓*, NO*↓	[26]
*E. histolytica* (ALA)	Curcumin	Nrf2↑, HO-1↑, NF-κB↓, IL-1*β*↓	[27]
*T. spiralis*	ALW–II–41-27	Nrf2↑, NF-κB ↓, TNF-α↓, IL-6↓, IL-17↓, ICAM-1↓	[28]
*T. spiralis*	Coptisine	Nrf2↑, HO-1↑, IL-1β↓, IL-18↓, TNF-α↓	[29]

GPx: glutathione peroxidase; SOD: superoxide dismutase; ROS: reactive oxygen species, NO: nitric oxide, DHA: Dehydroabietic acid, ZLE: *Ziziphus spina-christi* leaf extract, PPI: polysaccharide from Phellinus igniarius, IOP: Inonotus obliquus polysaccharide, GSH: glutathione, BZL: Benznidazole, SFN: sulforaphane, VEGF: vascular endothelial growth factor, DMF: Dimethyl fumarate, L. Inermis: Lawsonia inermis, iNOS: inducible nitric oxide synthase, ALA: amoebic liver abscess.

## 2 Malaria

Malaria is an acute febrile disease caused by *Plasmodium* parasites. It remains a potentially deadly disease. It is transmitted by a female *Anopheles* mosquito, either by inborn transmission or by transfusion of infected blood Products [30].

Red blood cells (RBCs) are the main cells infected by *Plasmodium* species. CD36, which is a receptor present in many cell types, mediating the internalization of *Plasmodium falciparum*-infected RBCs into macrophages. In phagocytes, it functions as a scavenger receptor for uptaking oxidized phospholipids and lipoproteins, and internalizing apoptotic cells and some pathogens; therefore, CD36 contributes to inflammatory responses [31]. Peroxisome proliferator-activated receptors, ligand 2, a nuclear receptor functioning as a transcription factor, can promote CD36 expression in macrophages. In inflammatory conditions such as malaria, cytokines like TNF-α and IFNγ decrease the expression of peroxisome proliferator–activated receptor γ (PPARγ), a nuclear receptor acting as a transcription factor and promoting CD36 in macrophages [32,33]. Macrophages with decreased CD36 on their surface have demonstrated remarkable phagocytic defects in confronting parasitized RBCs, as compared to wild-type macrophages. Surprisingly, macrophages lacking PPARγ did not display a complete loss of CD36. Accumulating evidence suggests that there might be alternative pathways promoting CD36 expression independently of PPARγ. The activated *Nrf2,* in response to oxidative stress, increases CD36 expression and promotes CD36-mediated *Plasmodium* phagocytosis. NRF2 is crucial in promoting CD36 expression and *Plasmodium* clearance in both *in vitro* and *in vivo* studies [33].

Pregnancy-associated malaria is a severe form of malaria infection associated with fetal growth restriction and a high risk of infant death. Sequestration of *Plasmodium falciparum*-infected erythrocytes in the placenta causes the recruitment of monocytes and macrophages to the placenta. This is associated with adverse outcomes [34,35]. CD36 is a scavenger receptor on monocytes and macrophages that serves several important protective roles, such as the destruction of malaria-infected RBCs. Among the *Nrf2*-regulated genes, HO-1 acts as a free heme detoxifier during malaria infection [36]. *CD36* expression is upregulated as a result of *Nrf2* stimulation. In addition, *Nrf2* mRNA level is inversely related to parasitemia, and *CD36* expression and *HO-*1 mRNA level are positively correlated with higher infant birth weights [37].

Disease tolerance to malaria is a kind of defense mechanism that particularly relies on renal proximal tubule epithelial cells (RPTEC) and does not target *Plasmodium* directly. This strategy is dependent on *HO-1* and ferritin H-chain (*FTH*) induction via NRF2.

The blood stage of Plasmodium spp. infection involves the parasite invading the host RBC and consuming up to 60–80 % of the RBC hemoglobin content. Plasmodium spp. lacks a gene for extracting iron from heme and, instead, acquires iron through heme auto-oxidation. When the infected RBCs are broken down, the remaining hemoglobin is released into the plasma [38]. Heme accumulation in the plasma and urine is associated with AKI, an indicator of severe malaria. *HO-1* and *FTH* expression in RPTEC is essential for the formation of disease tolerance to malaria. HO-1 catabolizes heme, and FTH stores iron in RPTEC. Therefore, HO-1 fails to establish disease tolerance to malaria alone, and it should be coupled with FTH. NRF2 can induce both HO-1 and FTH, consequently promoting disease tolerance to malaria [[38], [39], [40]].

**Fig. 3:**
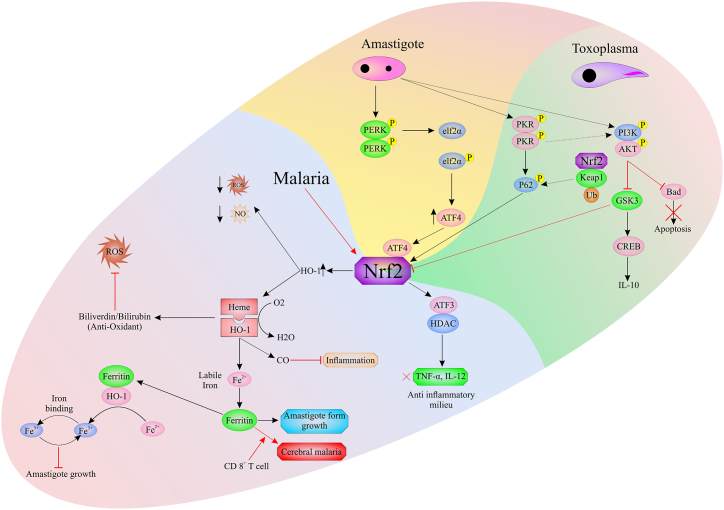
The Scheme of Nrf2 pathway in leishmaniasis, toxoplasmosis, and malaria. Upon exposure to amastigotes of *Leishmania*, the PERK/eIF2α/ATF4 signaling pathway activates, which resulted in the upregulation of ATF4 and HO-1. In toxoplasmosis, the PI3K-PKB/Akt pathway is the major route to prevent host cell apoptosis to evade from immune response, through phosphorylation of the pro-apoptotic BAD protein. Also, in toxoplasmosis and malaria, there is increased binding affinity between P62 and KEAP1, which is followed by free Nrf2 upregulation. ATF4: activating transcription factor 4, eIF2α: eukaryotic initiation factor-2 α, HO-1: Heme Oxygenase-1, KEAP1: Kelch-like ECH-associated protein, Nrf2: nuclear factor erythroid 2-related factor 2, PERK: PKR-like endoplasmic reticulum kinase.

Cerebral malaria (CM) is a severe malarial syndrome caused by *Plasmodium falciparum*. CM is associated with notable morbidity and mortality, despite anti-malarial treatment. Oxidative and inflammatory host responses and products released by *Plasmodium*-infected RBCs lead to brain endothelial cell activation and dysfunction [24]. The NRF2 pathway has a role in antioxidant and anti-inflammatory response regulation, showing favorable effects in cerebral vascular diseases, which share pathologic characteristics with CM. It is also a protective pathway in CM (Fig. 3). Therefore, it can be therapeutically activated in CM in order to achieve alleviative effects. Dimethyl fumarate is an FDA-approved drug upregulating NRF2 transcription and providing antioxidant and anti-inflammatory effects on endothelial cells [24,41]. Dimethyl fumarate treatment can reduce IL-6 and TNF-α in CM-derived parasite lines through several protective signaling pathways, including the NRF2 pathway [24].

*In this relation, Canavese* and colleagues investigated the effect of statins on the *Plasmodium berghei Anka mouse model* of cerebral *malaria.* The administration of Lovastatin had an upregulating effect on *TNF-α,* HO-1 and NRF2, as well as a downregulatory effect on INF-γ [25]. On the contrary, the treatment with simvastatin upregulated INF-γ and *TNF-α and did not have a* protective activity against cerebral *malaria. The combination administration of* lovastatin and vascular endothelial growth factor (VEGF) induced both HO-1 and NRF2, while preventing INF-γ and *TNF-α.* These findings, thus, show that VEGF-lovastatin treatment can prevent the *inflammatory response in the liver, spleen and brain* [25].

*Singh* et al. *also reported the antimalarial activity of ethyl acetate and fraxetin extracts from* Lawsoniainermis *(*L. Inermis*) plant in mice infected with* P. berghei*. It was also characterized that treatment with* L. Inermis *extracts significantly prevented the oxidative stress damage by stimulating NRF2,* superoxide dismutase (*SOD*), *Glutathione* (GSH)*, catalase (*CAT*),* glutathione S-transferases (*GST*), *an*d glutathione peroxidase (*GPx*)*. In addition, there was a significant reduction in the levels of inducible nitric oxide synthase (*iNOS*), NO and TNF-α* [26].

Cerebral malaria and severe malarial anemia (SMA) are known as severe malaria syndromes. SMA is the most common, and CM is the most lethal form of malaria [42]. It has been shown that NRF2 protects against experimental cerebral malaria by inducing HO-1 [43]. *Nrf2*-regulated genes are overexpressed in SMA, relative to CM, with the most expression in individuals with both SMA and HbSS. In other words, CM is associated with the downregulation of the NRF2 pathway, in comparison with SMA. There was no difference in HO-1 level between CM and SMA non-HbSS, but it was remarkably higher in people with SMA HbSS. These findings, thus, suggest that NRF2/HO-1 axis activation might contribute to protection from CM [43].

The spleen has a role in the removal of circulating malaria pathogens. Macroautophagy in splenocytes is a lysosome-associated process that degrades pathogens. Autophagy plays a crucial role in the regulation of the immune response, maturation of immune cells, and antigen presentation. NRF2 controls the expression of antioxidant genes and is also able to modulate *p62* expression. NRF2/KEAP1 is a complex disrupted in response to oxidative stress, which leads to the activation of NRF2. Consequently, NRF2 promotes the expression of antioxidant genes and, interestingly, *p62* expression [44,45]. So, by regulating *Nrf2*, *p62* can control its own expression. In advanced diseases, there is increased binding affinity between P62 and KEAP1. As a result, the elevated P62-KEAP1 interaction increases the free NRF2 level. It has been shown that the serine residues −349 and −403 positions in P62 are significantly more phosphorylated. The rise in serine phosphorylation at the 403rd position indicates that autophagy has been positively induced, while the increase at the 349th position shows that P62's affinity for KEAP1 has been raised (Fig. 3) [46].

## 3 Leishmaniasis

*Leishmania* parasite is typically transmitted to the mammalian host via infected sand fly saliva during blood-feeding, which is then internalized into phagocytes, predominantly macrophages [47]. Based on the infectious species, *Leishmania* spp. can primarily affect the skin and/or mucous tissue, which can be regarded as one of the factors determining the kind of cutaneous consequence and clinical implications, as well as influencing the immune system's inflammatory and anti-inflammatory responses [48]. In the host defense against intracellular parasitic infections such as leishmaniasis, macrophage cells quickly prompt oxidative stress responses for clearing the pathogens and trigger stress-related signaling cascades, regulating inflammation, and the innate and adaptive immunity [49,50].

NRF2 serves potential roles in cell protection from endogenous stressors and environmental insults. There is evidence indicating that this transcription factor regulates the expression of phase II defense genes that might defend cells against oxidative stress. It has crucial functions in anti-inflammatory and immune activator processes through Toll-like receptors [11,12,51].The NRF2 pathway decreases oxidative burst in *Leishmania* parasites and their cell hosts through the induction of anti-oxidative enzymes. Although the host cells can break this homeostasis, *Leishmania* parasites destabilize these signals in host cells and regulate the key *mediators* for the progression and establishment of infection [3].

*HO-1* is one of the *Nrf2* target genes contributing to heme metabolization and the production of free iron, carbon monoxide and bilirubin. HO-1 improves *Leishmania* viability by impeding the cytokines produced in inflammatory procedures. Activating transcription factor 3 (ATF3) is another target gene of *Nrf2*, which is upregulated in oxidative stress situations. *Atf3* is an adaptive-response gene serving vital functions in cellular procedures and the transduction of signals from different receptors to induce or inhibit the expression of downstream genes. In a recent study, Saha et al. showed that NRF2 counteracted with the oxidative burst-mediated host defense via upregulating *HO-1* expression. On the other hand, NRF2 upregulates *Atf3* expression and makes anti-inflammatory conditions essential for *Leishmania* survival. They confirmed that *Atf3* caused epigenetic regulation of IL-12 and TNF-α due to its function in histone deacetylase 1 (HDAC1) recruitment during *Leishmania* infection (Fig. 3). In addition, their results indicated that trigonelline hydrochloride, as an NRF2 inhibitor, could have potential therapeutic effects on visceral leishmaniasis (VL) in the *L. donovani*-infected mouse model [3,52,53]. In another study, Bichiou et al. revealed that *Leishmania* parasites increased NRF2, HO-1, Slc7a11, glutathione reductase (Gsr), CD36, and CAT expressions in C57BL/6 mouse bone marrow-derived macrophages (C57Bl/6 BMdMs). They showed that wortmannin treatment reduced the phosphorylation of the protein kinase B (*PKB*, or AKT)and the expression of HO-1 protein; in other words, PI3K/Akt activity is needed to enhance the expression of HO-1 during *Leishmania* infection [54].

Miltefosine is an analog of alkyl phosphocholine widely used in the treatment of different cancers, especially breast cancer. Miltefosine enhances the anti-*Leishmania* host immune response by increasing the number of T cells, WBCs, platelets, and macrophage-like cells. The anti-*Leishmania* effects of miltefosine depend on the induction of oxidative stress and the inhibition of Akt. NRF2 controls the innate immune responses by down-regulating nuclear factor-κappaB (*NF-κB*) expression and upregulating HO-1. HO-1 also modulates immune responses by influencing NRF2 translocation. Das et al. revealed that treatment of patients, affected by *Leishmania donovani* and *Leishmania infantum* as the main causes of visceral leishmaniasis, with miltefosine reduced the expression level of HO-1 and increased glutathione peroxidase, SOD, and ROS production. They also confirmed that miltefosine attenuated *Nrf2* expression in visceral leishmaniasis patients through the inhibition of extracellular signal-related kinase (ERK). Moreover, miltefosine inhibited IL-10 and TGF-β expression and induced IL-12 and TNF-α expression by reducing HO-1 [[13], [14], [15]]. Taken together, these findings suggest that targeting NRF2/HO-1 signaling should be considered as a topic of research to treat leishmaniasis. Quercetin is a natural compound that can act as an antiprotozoal, antiviral, antibacterial and antioxidant in human cells. It can also complement antimonial therapy and reduce the side effects of the drugs used in *Leishmania* treatment [55]. Further, Cataneo et al. declared that quercetin decreased the viability rates of *L. braziliensis* promastigotes via upregulation of *Nrf2* and *HO-1* expressions. This compound not only increases NRF2/HO-1 and IL-10, but also decreases TNF-α [16]. Dehydroabietic acid (DHA) is a member of the resin acid family that is extracted from Pinuselliottii and has anti-inflammatory, antimicrobial, and antifungal effects. According to recent studies, DHA serves potential roles as an anti-protozoan against *L. braziliensis*, *L. infantum*, *L. donovani*, and *Trypanosoma cruzi* [56,57]. Goncalves et al. displayed that DHA reduced the proliferation of *L. amazonensis* promastigotes due to the down-regulation of NRF2/ferritin expression, an increase in free iron and iron bound to transferrin, and ROS production. They found that DHA decreased the percentage of infected macrophages and amastigotes per macrophage without changing the viability of macrophages [17].

Another NRF2-related pathway is the PERK/eIF2α/ATF4 axis, which has a vital function in the cell survival against nutritional, hypoxic, and oxidative stresses. *PKR-like endoplasmic reticulum kinase (PERK*) increases the expression of antioxidative genes by triggering the nuclear factor NRF2 [58,59]. In this regard, Dias Teixeira et al. demonstrated that infection with *L. amazonensis* activated PERK/eIF2α/ATF4 signaling pathway in the human tissue and cultured macrophages. They also indicated that the expression of *Atf4* and *HO-1* was upregulated due to *L. braziliensis* infection. In addition, the available evidence confirmed that the down-regulation of PERK or ATF4 attenuated NRF2 and HO-1 expression and enhanced nitric oxide production [60].

It has been shown that Glycogen synthase kinase 3 (GSK3) is a target gene of the PI3K/Akt signaling pathway that phosphorylates the Nh6 domain of NRF2, which is followed by proteasomal degradation of NRF2 when infected with *Leishmania amazonensis* [61]. P62 phosphorylation leads to its binding to KEAP1and causes autophagy via upregulation of NRF2 expression (Fig. 3) [62]. On the other hand, the protein kinase R (PKR) stimulates autophagy via the phosphorylation of eukaryotic initiation factor-2 α (eIF2α) [63]. In addition, Vivarini et al. established that NRF2- or PKR/Akt-deficient macrophages resulted in the upregulation of ROS/RNS, the down-regulation of *Sod1*, which is an *Nrf2*-dependent gene, and an attenuated parasite load. Furthermore, *L. amazonensis* counteracted *Keap1* through the upregulation of *p62* via *PKR* [64].Central signaling cascades such as PERK, PI3K/Akt and *PKR* are important parts of regulating the NRF2 antioxidant pathway in leishmaniasis [3]. Overall, these results demonstrated that PI3K/Akt and NRF2/PKR crosstalk pathways could act as potential targets in human cutaneous leishmaniasis treatment.

## 4 Trypanosomiasis

**Fig. 4:**
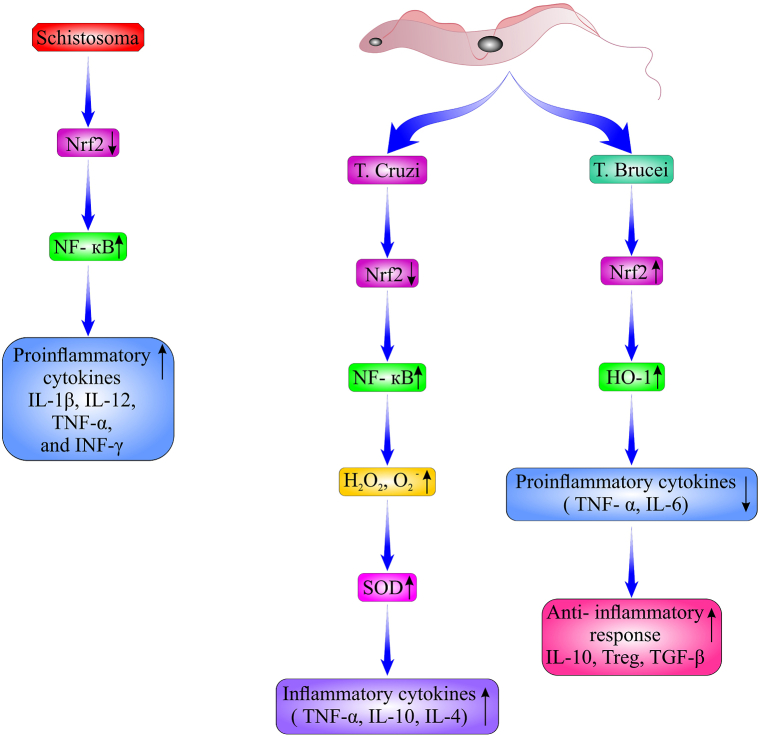
The Scheme of Nrf2 pathway in schistosomiasis*, T. cruzi and T. brucei*. In schistosomiasis, downregulation of Nrf2 results in the upregulation of NF-κB and subsequently activation of proinflammatory cytokines. In *T. cruzi* infection, increased oxidative stress was accompanied by Nrf2 downregulation and NF-κB upregulation. In addition, the production of H_2_O_2_ and O_2_^−^ was induced by *T. cruzi* infection. Together, these changes were accompanied by increased inflammatory cytokines. *T. brucei* infection can induce the Nrf2/HO-1 pathway and suppress the host immune inflammatory response. HO-1: Heme Oxygenase-1, H_2_O_2_: hydrogen peroxide, NF-κB: nuclear factor-κappaB, Nrf2: nuclear factor erythroid 2-related factor 2, O2−: superoxide anion, SOD: superoxide dismutase, *T. cruzi: Trypanosoma cruzi, T. brucei*: *Trypanosoma brucei*.

*Trypanosoma cruzi*, the causative agent of Chagas disease, is a protozoan affecting millions of people all over the world [65,66]. The transmission of *T. cruzi* occurs through triatomine insect bites, blood transfusions, and congenital and organ transplantation [67]. Oxidative stress contributes to the tissue damage during the acute Chagas infection. Increased oxidative stress parameters and apoptosis levels are accompanied by NRF2 downregulation and NF-κB upregulation. The production of hydrogen peroxide (H_2_O_2_) and superoxide anion (O_2_^−^) is induced by *T. cruzi* infection [20]. Studies on melatonin, as an anti-inflammatory/antioxidant hormone, in *T. cruzi*-infected rats have demonstrated that it can attenuate H_2_O_2_ and O_2_^−^ production. These changes are also accompanied by increased NRF2 and decreased NF-κB, IL-10, IL-4 and TNF-α (Fig. 4) [20].

Although various parasites are susceptible to oxidative stress as a defense mechanism, some, such as *Trypanosoma cruzi,* resist the oxidative environment [68,69]. By investigating the impact of ROS in *T. cruzi* infection using mice deficient in ROS production and parasites with enhanced DNA repair enzymes, the researchers have found that ROS not only plays a role in pathogen defense, but also acts as a signaling molecule promoting *T. cruzi* growth within host cells [[70], [71], [72]]. In another study focusing on the therapeutic effects of HO-1 induction in *T. cruzi* infection, Claudia N. Paiva et al. revealed that NRF2/HO-1 induction increased resistance to infection by *T. cruzi*, decreased *T. cruzi* burden, and raised macrophage parasitism. They also showed that oxidative stress favored *T. cruzi* parasitism and promotes its growth [71]. In one other study, Florentino et al. infected HeLa cells and AC16 human cardiomyocyte cell line with *T. cruzi,* showing that NRF2 expression could be upregulated at early stages of infection and was reduced at the later stages. Collectively, these data confirm that antioxidant reduction may be beneficial for the multiplication of parasites and sustainment of the infection. They also verified that NRF2induction by Benznidazole (BZL) and sulforaphane, as an NRF2 activator, inhibited the growth of intracellular parasites and reduced the parasite burst in these cells [22]. Another *in vitro* study also showed that BZL had a trypanocidal effect on *T. cruzi*, leading to the increase of NRF2 and GSH [23].

One of the most pathogenic species of *Trypanosoma* sp. is *T. brucei*, an exclusively extracellular protozoan parasite. In the mammalian host, the blood and central nervous system (CNS) are the most important places for *T. brucei* to live [[73], [74], [75]]. *HO-1* expression can lead to the host immune response reduction and parasite persistence [[76], [77], [78]]. Previous investigations have reported *HO-1* upregulation in some parasitic infections, such as *Plasmodium*, *Fasciola hepatica*, and *Leishmania chagasi* [76,77,79]. Nicole K. Campbell et al. have also revealed that *T. brucei* secretes some aromatic ketoacids followed by NRF2 activation and, subsequently, upregulation of HO-1 in glia and macrophages. This induction of the *Nrf2*/*HO-1* pathway results in host immune suppression. They have also demonstrated that these *trypanosome*-derived aromatic ketoacids reduce IL-6 and TNF-α production in glial cells [74].

## 5 Toxoplasmosis

*Toxoplasma gondii* is an obligate intracellular parasitic protozoan that causes toxoplasmosis in warm-blooded animals, like humans. Toxoplasmosis is an infection with high prevalence worldwide that can be life-threatening, especially in immunocompromised persons in congenital forms [[80], [81], [82], [83], [84]]. Human infections are usually acquired by the consumption of viable tissue cysts in contaminated raw meat or *T. gondii* oocysts in food or water. Many infections in adults are mainly asymptomatic, but ocular or lymphadenopathy manifestations can occur in some patients [85].

The liver is one of the most important organs attacked and damaged by *T. gondii*. It can cause hepatomegaly, hepatitis, and cirrhosis in an infected liver [[86], [87], [88]]. The main mechanism of the liver damage in toxoplasmosis is inflammatory responses. Pro-inflammatory cytokines like TNF-α, IL-1β, and IL-6 have important roles in this process. Since these cytokines are regulated by NF-κB signaling, inhibiting this pathway can reduce *T. gondii*-induced liver inflammation [[89], [90], [91]]. Therefore, oxidative stress plays an important role in *T. gondii*-induced liver injury [92]. NRF2 activation reduces oxidative stress and inflammation by upregulating some enzymes with antioxidative function [78,[93], [94], [95]]. A recent study has indicated that the accumulation of P6 protein, in cells infected with *T. gondii*, activates the NRF2 signaling pathway. Ubiquitination and degradation of KEAP1 are induced by P62-mediated autophagy which are*,* followed by the activation of the NRF2/HO-1 pathway [96]. PI3K-PKB/Akt pathway is another toxoplasmosis-related signaling discussed in detail in the *Leishmania* section. This signaling pathway is identified as one of the major inhibitors of apoptosis during *T. gondii* infection by phosphorylation of the pro-apoptotic BAD protein (Fig. 3) [97,98].

Sulfadiazine and pyrimethamine are the most effective available therapeutic agents used to treat toxoplasmosis. While these drugs can cause serious side effects, researchers are looking for new alternatives that are both effective and less toxic [81,99]. Inonotus obliquus polysaccharide (IOP) is one of these newly considered alternatives with both antioxidative and anti-inflammatory activities [92,[100], [101], [102], [103], [104], [105]]. Lu Xu et al. revealed that IOP had hepatoprotective activity in mice infected by *T. gondii*. They also reported that one of the mechanisms of IOP-mediated hepatoprotection was its antioxidative and anti-inflammatory activity by increasing the expression of *Nrf2*/*HO-1* [89]. Other complications of *T. gondii* infection are reproductive dysfunction and a decline in sperm quality and male fertility [106,107]. *T. gondii*-induced oxidative stress and apoptosis of testicular cells are the main causes of these complications [108,109]. NRF2 activation can inhibit *T. gondii*-induced testicular oxidative damage by upregulating antioxidant gene transcriptions like HO-1 [110,111]. Xiao ding et al. also demonstrated that IOP resulted in spermatogenesis improvement in mice by reducing the oxidative activity and enhancing the antioxidative activity. In this study, they also demonstrated that IOP increased the expression of testicular *Nrf2* and *HO-1*, which suggested that the antioxidative effects of IOP could be related to the activation of the NRF2 signaling pathway [111]. These findings not only provide information to further investigate the mechanism of the oxidative stress during *T. gondii* infection, but also indicate the clinical applications of drugs affecting these pathways.

ROS plays an important role in innate immunity against toxoplasmosis [112,113]. The host defense system increases its intracellular oxidative stress by increasing the production of ROS to resist *T. gondii* invasion [[114], [115], [116], [117], [118]]. The NRF2 pathway can reduce and balance this oxidative stress. Yu pang et al. showed that NRF2 signaling was very important for *T. gondii* survival in the activated macrophages of mice. They also demonstrated that the *T. gondii* proliferation was repressed in the activated NRF2-deficient cells in mice [96]. Although most parasites are eradicated in immunocompetent hosts, some of them are differentiated into slowly replicating forms (bradyzoites), escaping host immune responses and making tissue cysts in the brain and muscle [119]. Previous studies have also shown that the microenvironment of the host cells plays an important role in triggering bradyzoite formation. For example, oxidative stress increment in myotubes triggers bradyzoite formation [[120], [121], [122], [123], [124]]. Further, Rahman et al. demonstrated that higher oxidative stress and lower *Nrf2* transcriptional activity in myotubes could trigger bradyzoite formation and, reversely, lower oxidative stress and higher *Nrf2* transcriptional activity in myoblasts could inhibit bradyzoite formation [124].

## 6 Schistosomiasis

Schistosomiasis is a parasitic disease caused by flatworms of the genus *Schistosoma*. This genus is composed of trematode flatworms with three main species, including *Schistosoma haematobium*, *Schistosoma japonicum* and *Schistosoma mansoni*. They can involve various organs of humans, such as the intestine, bladder, liver, brain, and spinal cord, and may cause significant morbidity or, in some cases, death [125,126].

One of the important disorders reported for schistosomiasis is neurological impairment, which is observed in children as learning disorders and memory deficits, and in adults as a short attention span, impaired work capacity, and cognitive deficits [127,128]. However, while there is not much information regarding the mechanisms of schistosomiasis-induced neurological impairments, it seems that systemic inflammation is a major origin of neuroinflammation in schistosomiasis and the subsequent neurologic signs and symptoms [129,130]. In a recent study, Gasparottoet al. demonstrated that systemic infection with *S. mansoni* in a murine model without any parasite invasion of CNS led to astrocyte and microglia activation, oxidative damage, and expression of the oxidative stress-induced transcription factor NRF2. This protein can control the innate immune responses by down-regulating *NF-κB* expression and upregulating *HO-1*. Predominantly, in schistosomiasis, downregulation of NRF2 results in the upregulation of *NF-κB,* which is followed by the activation of proinflammatory cytokines (Fig. 4). In addition, other proinflammatory cytokines, including MCP-1, IL-12, TNF-α and IFN-γ, were significantly increased in the *S. mansoni*-infected *mouse serum* [129].

Another serious manifestation of *S. mansoni* infection is liver fibrosis, which is caused by eggs accumulation. In this regard, Almeer et al. reported that NRF2 expression was downregulated in infected mice with *S. mansoni;* on the other hand, *Ziziphusspina-christi* leaf extract could inhibit liver fibrosis by modulating the expression of NRF2, *TGF-β*, *α-SMA*. Further, *S*. *mansoni* infection induced the expression of the pro-inflammatory cytokines *COX-2*, *IL-1β*, *α-SMA* and *TNF-α* [18].

Infection with *Schistosoma japonicum* leads to liver fibrosis, which is called schistosomiasis-associated liver fibrosis (SSLF) [131]. The main causes of SSLF are the formation of egg granulomas in the liver, and the activation and proliferation of hepatic stellate cells (HSCs) [132]. Cannabinoid 1 (CB1) receptor signaling has profibrogenic effects, and CB1 suppression can inhibit HSCs activation [133]. In a study on mice livers, Wang et al. confirmed that CB1 upregulation played a role in the development of SSLF. They also revealed that NRF2 activation led to *CB1* upregulation and *Nrf2* deactivation inhibited *CB1* by soluble egg antigens (SEA) of *S. japonicum* in HSCs [134]. Overall, these findings seem to imply that NRF2 has a detrimental effect on the development of SSLF.

Jia-Ning et al. designed a mouse model of *S. japonicum* cercariae infection in which the parasites developed hepatic fibrosis and granuloma. *S. japonicum* infected mice exhibited increased serum malondialdehyde levels and a decrease in antioxidant enzymes and glutathione. In addition, they showed that the treated mice with polysaccharide from *Phellinus igniarius* (PPI) presented a reduction in collagen deposition and hepatic egg granulomas, as compared to the infected non-treated mice. Furthermore, PPI treatment could play an effective role in improving oxidative stress damages by upregulating *Gsta4*, *GSH* and *Nrf2,* and downregulating *TGF-β*gene expression [135].

## 7 Entamoebiasis

*Entamoeba histolytica* (*E. histolytica*) is a harmless commensal protozoa that can be transmitted by the ingestion of contaminated water or food containing infective cysts [136].

Although most cases of infection are asymptomatic, intestinal invasion may manifest itself with several weeks of abdominal pain, cramping, bloody or watery diarrhea, and loss of weight. Disseminated and Extra-intestinal amoebiasis such as pneumonia, amoebic liver abscess (ALA), and cerebral amoebiasis have been reported too [136].

**Fig. 5:**
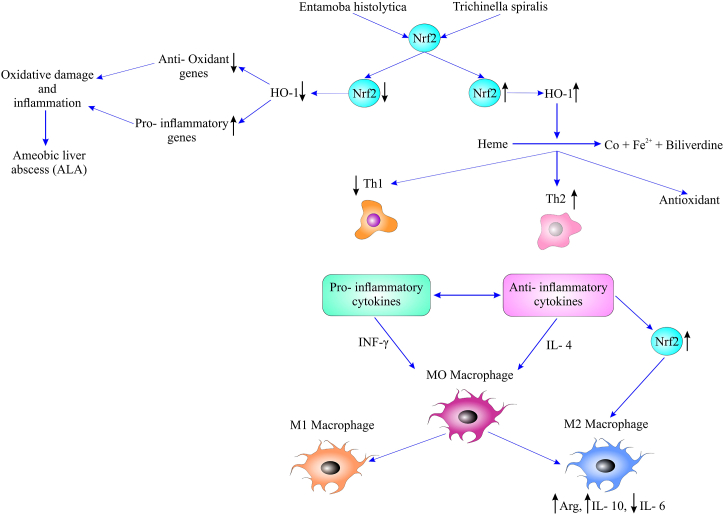
The role of Nrf2 pathway in entamoebiasis and trichinosis. During ALA, after the downregulation of Nrf2 and HO-1, the antioxidant gene expressions are reduced while the pro-inflammatory genes are activated, resulting in uncontrolled oxidative damage and inflammation. Furthermore, the role of Nrf2 in the development of polarized macrophages has been indicated in trichinosis. Arg: Arginase-1, ALA: amoebic liver abscess, HO-1: Heme Oxygenase-1, Nrf2: nuclear factor erythroid 2-related factor 2.

This parasite primarily infects the colon, but it can migrate to the liver as a result of colon perforation, leading to ALA with substantial mortality and morbidity. ALA, which is the most prevalent extra-intestinal complication of *E. histolytica*, is characterized by one or several abscesses [137]. Inflammatory responses and oxidative stress are the important factors in the pathogenesis of ALA. During ALA, the host antioxidant mechanism of NRF2 is downregulated, while the pro-inflammatory pathway is activated, resulting in uncontrolled oxidative damage and inflammation (Fig. 5). These data, thus, suggest that NRF2 and its inducers can be an alternative to prevent or optimize ALA treatment [138,139].The effects of curcumin (a bioactive component of turmeric) on ALA in hamsters have been studied, suggesting that curcumin treatment can prevent the activation of NF-κB and IL-1β, while it increases HO-1 expression. Therefore, the overexpression of *HO-1* indicates the activation of NRF2 [27]. In a different study, Aldaba-Muruato and colleagues investigated the relationship between ALA and the adrenergic system in the hamster. Phentolamine (α-adrenergic blocking agent) and propranolol (β-adrenergic *blocking* agent) treatments reduced oxidative stress and the proinflammatory system (IL-1β and NF-κB). In addition, phentolamine treatment increased NRF2 and HO-1 expression levels. These findings, thus, imply that oxidative stress and NRF2 signaling pathways may be involved in adrenergic system regulation during ALA infection [140].

Despite all these efforts, there are no therapeutic or prophylactic medications to prevent amoebiasis. Prevention of fecal-oral exposure, disinfection of vegetables and water, and hand hygiene are the most frequently used methods for prevention [27]. However, these prevention measures are not the most effective in developing countries or under environmental disaster conditions. The effective compounds on the NRF2 signaling pathway may be regarded as alternative hepatoprotective agents to prevent amoebic liver abscess.

## 8 Trichinosis

*Trichinella spiralis* (*T. spiralis*) is an intracellular nematode worm that is the causative agent of trichinosis. The T. spiralis life cycle is confined to a single host species, and muscle, enteric, and larval stages are three main life phases [141]. It is transmitted to human through the ingestion of infected meat (horses, wild and domestic pigs) containing the encysted larval stage [142]. In humans, trichinosis has been linked to some infectious and non-infectious diseases, such as Crohn's disease, Irritable bowel syndrome, and RSV (respiratory syncytial virus) infection [[28], [143], [144]]. Here, we evaluate the role of NRF2 in trichinosis-related disorders.

Crohn's disease is a chronic inflammatory disease of the gastrointestinal tract. It is associated with increased Th1 cells, which can produce pro-inflammatory cytokines and oxidative stress [145,146]. It seems that macrophages are the main target of treatment for Crohn's disease. The Th2 cell response is mediated by the polarization of alternately activated macrophages (M2), which can suppress the Th1 cell response [147,148]. Th2 cells then promote anti-inflammatory responses via IL-4 and IL-10 [[149], [150], [151]]. Excretory/secretory (ES) products from *Trichinella spiralis* muscle larvae may induce M2, which helps relieve colitis [152]. NRF2 can decrease uncontrolled inflammation by promoting antioxidant responses and activating M2 polarization [153,154]. Jin et al. also investigated the role of NRF2 in the development of macrophages polarized by *Trichinella spiralis*. In this study, M2 polarization was induced by the ES products of *Trichinella spiralis in vitro*. Macrophages of wild-type mice showed increased levels of Arginase-1 and anti-inflammatory cytokine IL-10. Conversely, macrophages from *Nrf2* knockout (KO) mice showed decreased levels of Arginase-1 and anti-inflammatory cytokine IL-10. This, thus, indicates that NRF2 is involved in M2 polarization induced by *Trichinella spiralis*. Moreover, *Nrf2* KO macrophages treated with ES products failed to improve colitis induced by TNBS (a well-established model of human Crohn's disease) *in vivo*. Instead, macrophages from wild-type mice treated with ES products alleviated the severity of inflammation, which indicated the significance of NRF2 in M2 polarization (Fig. 5). Compared to the TNBS group, Th1 cells in the ES-M, rather than the ES-M (KO) treatment group, were significantly reduced and the number of Th2 cells was considerably increased. This reveals the critical role of NRF2 in regulating cytokine production [143].

Irritable bowel syndrome (IBS) is a functional GI disorder characterized by recurrent pain. Some patients with IBS develop post-infectious IBS (PI-IBS) following acute GI infection [155]. Inflammation and oxidative stress are linked to the development of PI-IBS [156]. Hepatocellular tyrosine kinase receptors that produce erythropoietin are crucial for contact-dependent cell communication, oxidative stress, the inflammatory response, and IBS. EphA2, a member of the Eph receptor family, influences various signaling pathways, including NRF2 and NF-κB, inducing oxidative stress and inflammatory responses [151,157]. According to a study done by Zeng et al., EphA2 levels were increased in human intestinal epithelial cells treated with LPS, and in mice infected with *Trichinella spiralis*. In the colon of the mice infected with *Trichinella spiralis*, the nuclear NF-κB/p65 abundance was greatly enhanced, whereas the nuclear translocation of NRF2 was dramatically reduced. In order to evaluate the effects of ALW–II–41-27 (an EphA2 inhibitor), *Trichinella spiralis*-infected mice were used. Treatment with ALW–II–41-27 resulted in considerably higher levels of the nuclear NRF2, as compared to the PI-IBS group, and noticeably lowered the levels of NF-κB nuclear translocation in the colon of ALW–II–41-27-treated animals. As a result, ALW–II–41-27 had the ability to reduce inflammation and oxidative stress *in vivo* [28].

A recent study showed that Coptisine treatment, an isoquinoline alkaloid isolated from *Coptischinensis*, decreased the scores of *abdominal withdrawal* reflex and gastrointestinal motility in the *T. spiralis*-infected PI-IBS rat model. In this study, it was revealed that coptisine treatment suppressed the level of proinflammatory cytokines (IL-1β, IL-18, and TNF-α) and oxidative stress markers. Furthermore, coptisine significantly induced *Nrf2* and *HO-1* expression [29]. Given the potential protective effects of coptisine against PI-IBS, we can consider a therapeutic agent for this disease.

Acute lower respiratory infections are often caused by RSV, which has a high mortality rate [158]. Oxidative stress is one of the factors contributing to the severity of the inflammatory response during an RSV infection. RSV downregulates NRF2, thus reducing the expression of antioxidant enzymes [159]. In another study, the effects of *Trichinella spiralis* infection on RSV-infected mice were assessed. Despite a striking rise in NQO1, an antioxidant enzyme, a single infection with *T. spiralis* had no impact on NRF2. On the other hand, a single RSV infection led to decreased NRF2. Increased NRF2 and NQO1 expression was observed in the Ts-RSV co-infected mice. The IgM and IgG antibody responses to RSV were increased by *T. spiralis* infection. It raised IgA levels as well. *T. spiralis* decreased the amount of the RSV virus in the lung tissue. In the lung tissue, it decreased the RSV viral load as well. Additionally, compared to the RSV control group, it significantly decreased the cellular inflow in the bronchoalveolar lavage fluid in the Ts-RSV group [144].

In another study done by Salama et al., the effects of zinc and resveratrol on the anti-helminthic, anti-inflammatory, and antioxidant activities were studied during *T. spiralis* infection. *Treatment with* these agents resulted in a reduction of parasite load, malondialdehyde level, and a significant increase in NRF2, IL-12, and the total antioxidant capacity (TAC) [160].

## 9 Conclusion

Given the molecular intricacy distinguishing parasite-host cell interactions, it is difficult to categorize a factor like NRF2 as having a positive or negative effect in the course of a parasitic infection. Overall, by inducing the antioxidant and anti-inflammatory response, NRF2 has a dual function in parasitic infection that can benefit both the parasite and the host. *Trypanosoma cruzi* and other ROS-resistant parasites may be damaged by a strong antioxidative state brought on by NRF2 activation, which can also lessen their burden on the host. On the other hand, it has been confirmed that suppressing NRF2 expression can offer therapeutic benefits in visceral leishmaniasis. To understand the molecular basis of parasite infections and to apply efficient therapeutics, either boosting or inhibiting NRF2, it is crucial to distinguish such a behavior in various contexts. As a result, it may be important to investigate how various signaling pathways might activate or inhibit NRF2 when creating novel treatment approaches for parasitic diseases.

**Table:** Abbreviation table

ALA	Amoebic Liver Abscess
ATF3	Activating Transcription Factor 3
BZL	Benznidazole
CB1	Cannabinoid 1
CM	Cerebral Malaria
DHA	Dehydroabietic Acid
eIF2α	Eukaryotic Initiation Factor-2 α
ERK	Extracellular Signal-Related Kinase
ES	Excretory/Secretory
FTH	Ferritin H-Chain
GSH	Glutathione
Gsr	Glutathione Reductase
GPx	Glutathione Peroxidase
GST	Glutathione S-Transferases
GSK-3	Glycogen Synthase Kinase 3
HO-1	Heme Oxygenase-1
IFNγ	Interferon γ
iNOS	Inducible Nitric Oxide Synthase
IOP	Inonotus Obliquus Polysaccharide
Keap1	Kelch-like ECH-Associated Protein
KO	Knockout
L. Inermis	Lawsonia Inermis
NQO1	NAD (P) H Quinone Dehydrogenase 1
NF-κB	Nuclear Factor-κappaB
Nrf2	Nuclear Factor Erythroid 2-Related Factor 2
PERK	PKR-Like Endoplasmic Reticulum Kinase
PKB	Protein Kinase B (Akt)
PKR	Protein Kinase R
RPTEC	Renal Proximal Tubule Epithelial Cells
SOD	Superoxide Dismutase
SMA	Severe Malarial Anemia
SSLF	Schistosomiasis-Associated Liver Fibrosis
VEGF	ascular Endothelial Growth Factor

## Ethics approval and consent to participate

This research was supported by the 10.13039/501100006662Ardabil University of Medical Sciences (grant number: IR.10.13039/501100006662ARUMS.REC.1401.226).

## Consent for publication

Not applicable.

## Funding

None

## Data availability statement

The data pertaining to this study have not been deposited in a publicly accessible repository, given that all relevant data are thoroughly detailed in the article, supplementary materials, or appropriately cited in the manuscript.

## CRediT authorship contribution statement

**Mohammadamin Vatankhah:** Writing – original draft, Investigation. **Reza Panahizadeh:** Writing – original draft, Investigation. **Ali Safari:** Investigation, Data curation. **Alireza Ziyabakhsh:** Investigation. **Behnam Mohammadi-Ghalehbin:** Investigation. **Narges Soozangar:** Writing – review & editing, Writing – original draft, Investigation. **Farhad Jeddi:** Writing – review & editing, Writing – original draft, Investigation.

## Declaration of competing interest

The authors declare that they have no known competing financial interests or personal relationships that could have appeared to influence the work reported in this paper.
